# Assessment of Debris Extrusion in Curved Canals: An In Vitro Analysis of Various Single-File Endodontic Instrumentation Systems

**DOI:** 10.1155/2024/8367693

**Published:** 2024-07-05

**Authors:** Muhammad Zubair Ahmad

**Affiliations:** Department of Conservative Dental Sciences College of Dentistry Qassim University, Buraydah 52571, Saudi Arabia

## Abstract

**Objectives:**

Rotary single-file endodontic systems are commonly used for root canal treatment. However, very few studies have evaluated the apical extrusion of debris generated by these systems during canal preparation at normal body temperature in laboratory conditions. The aim of this study was to evaluate the amount of apically extruded debris caused by seven different single-file endodontic instrumentation systems at body temperature in mandibular molar teeth with curved root canals.

**Methods:**

One hundred forty mandibular first permanent molars were randomly divided into seven groups (*n* = 20) to be prepared by one of the following systems at 35°C: Reciproc Blue (REC Blue), WaveOne Gold (WOG), One Reci (OR), Neoniti, HyFlex EDM (HEDM), One Curve (OC), or XP Shaper (XPS). Debris was collected into preweighted Eppendorf tubes. The weight of the extruded debris was recorded by subtracting the weight of the tooth-free apparatus from the post-procedure weight. Data were analyzed by one-way ANOVA and Tukey's tests (*p*  < 5%).

**Results:**

REC Blue, WOG, and OR groups extruded significantly more debris from the apex than XPS, OC, and Neoniti groups (*p*  < 0.05). No significant difference was observed among the XPS, OC, Neoniti, and HEDM groups (*p*  > 0.05).

**Conclusion:**

All the instruments were associated with debris extrusion. However, REC Blue, WOG, and OR extrude significantly more debris than other instruments. The amount of debris with different files was REC Blue > OR > WOG > HEDM > OC > Neoniti > XPS. XPS, Neoniti, and OC caused significantly less extrusion of debris than REC Blue, WOG, and OR.

## 1. Introduction

A considerable part of various dental treatments consists of root canal treatment permitting patients to preserve their teeth. The very high success rate of root canal treatment comparable to dental implants [1, 2] is noteworthy; despite this high success rate, complications during and after treatment can occur. For instance, during root canal instrumentation, pulp tissues, microorganisms, irrigants, and dentine chips may be expelled through the apex into the periradicular tissues [3]. Postoperative complications and pain may arise because of apically extruded debris and such complications are reported in literature ranging from 1.4% to 16% [4].

Endodontic instruments have been evolving continuously from hand instruments to motor-driven multiple and single-file systems as a result of research and industrial input [5]. Extrusion of debris and irrigants beyond the apex remains a challenge, even with various root canal preparation techniques and improved designs of root canal instruments. Continuous rotation and reciprocation are two main types of motions that can be used to classify single-file endodontic instrumentation systems [6].

Al-Saffar and Al-Gharrawi [7] found R-Motion (RM; FKG Dentaire, La Chaux-de-Fonds, Switzerland) single-file system produced significantly less debris extrusion as compared to standard single-file reciprocating and multifile rotary systems in moderately curved canals of maxillary first molars. Mehra et al. [8] compared single and multifile endodontic instrumentation systems and found that TruNatomy (TN; Dentsply Sirona, Ballaigues, Switzerland) files produced less debris extrusion than other files in mandibular premolars with single canals. The difference, however, was not statistically significant (*p* > 0.05). De Moura et al. [9] compared Reciproc (VDW, Munich, Germany) with WaveOne Gold (WOG; Dentsply Sirona Endodontics, Tulsa, OK, USA) to evaluate apically extruded debris in mesial roots (from 10° to 20°curvature) of mandibular first molars and found no difference between both file systems when canals were prepared at different working lengths.

Despite the fact that useful knowledge on apical extrusion of debris during root canal instrumentation exists, there is insufficient information regarding debris extrusion due to single-file instrumentation systems in canals with severe curvature, and more research is needed to compare novel instruments [10]. Reciproc Blue (REC Blue, VDW, Munich, Germany) is an updated version of Reciproc (VDW, Munich, Germany), a single-file system that works in a reciprocating motion. WaveOne Gold (WOG; Dentsply Sirona Endodontics, Tulsa, OK, USA) is a single-file system that is heat-treated and works in a centric reciprocation type of motion. XP-endo Shaper (XPS; FKG Dentaire SA, La Chaux-de-Fonds, Switzerland) is the single-file system used in a full rotation motion. HyFlex EDM (HEDM; Coltene/Whaledent, Altstätten, Switzerland) and Neoniti A1 (Neolix, Châtres-la-Forêt, France) are the single-file, rotary NiTi instruments that work in a continuous rotational motion in the clockwise direction. One Reci (OR; MicroMega, Besancon, France) is a recently launched single-file system that works in a reciprocating manner. Another single-file system is One Curve (OC; MicroMega, Besancon, France), which has the same design characteristics as OR; other than that, it works in a continuous rotation manner.

The degree of curvature and highly variable anatomy of the root canals of molars not only increases the difficulty level of instrumentation but also influences the extrusion of debris apically [11, 12]. To date, using different kinematics, a very small number of studies have examined the debris extruded apically from the curved canals [5, 10, 13].

To our knowledge, no study is available comparing the effect of the aforementioned files on the quantity of debris extruded apically in curved canals. Studies that compare different file systems used room temperature settings. Because XPS expands at body temperature and changes its shape, a reliable conclusion cannot be drawn using conventional methods to quantify apically extruded debris at room temperature settings in the laboratory. Hence, the objective of the present study was to quantify and compare the debris extruded apically by XPS, HEDM, REC Blue, WaveOne Gold, Neoniti, One Reci, and OneCurve during the preparation of the root canal at body temperature using a modified measuring model. The null hypothesis tested was that there would be no difference among XPS, HEDM, REC Blue, WaveOne Gold, Neoniti, One Reci, and OneCurve regarding apically extruded debris.

## 2. Materials and Methods

The study protocol was approved by the Institutional Review Board (IRB) of Qassim University, Saudi Arabia (registration no. 21-21-08).

Root surfaces of selected teeth were debrided using the method described by Al-Saffar and Al-Gharrawi [7]. The specimens were immersed for 24 hr in a 1% sodium hypochlorite solution at 4°C, after which specimens were stored in a saline solution [14, 15].

### 2.1. Sample Selection

We used G Power 3.1.9.2 software (Heinrich Heine University, Dusseldorf, Germany) for sample size estimation based on a previous study [16]. Seven groups of 20 teeth were formed to achieve 80% power and a 0.05 *α* error of probability. One hundred and forty mandibular first permanent molar teeth having a total length between 21 and 23 mm, which were extracted for periodontal reasons and had fully formed apices, were selected. Inclusion criteria were teeth without previous endodontic treatment, with mature and patent apices. For the selection of root specimens, we took radiographs of each tooth and measured the curvature angles with image analysis software (Adobe Photoshop CS3; Adobe Systems Inc, San Jose, CA). In this study, we included teeth with root angles from 20° to 40° [17] and with < 10 mm radii of curvature as described by Schafer et al. [18]. We discarded the distal root after sectioning it with the respective part of the crown at the level of furcation using a diamond saw (Isomet 1000; Buehler Ltd., Lake Bluff, IL, USA) at low speed. We used a round carbide bur (Diatech, Coltene Whaledent, Alstätten, Switzerland) at high speed under cooling water to prepare the endodontic access cavities. A high-speed bur was used to flatten the crowns of all teeth to ensure standardization and to obtain a reference point. The lengths of all teeth were standardized to 19 mm. Any tooth with an apical diameter greater than size 10 was excluded. A barbed broach (VDW) was used to remove the pulp remnants. To find the working length (WL), we inserted a size 10 K file (Dentsply Maillefer) into the canal until it was visible at the apical foramen. We then subtracted 1 mm from this length to get the WL.

### 2.2. Test Apparatus

**Figure 1:**
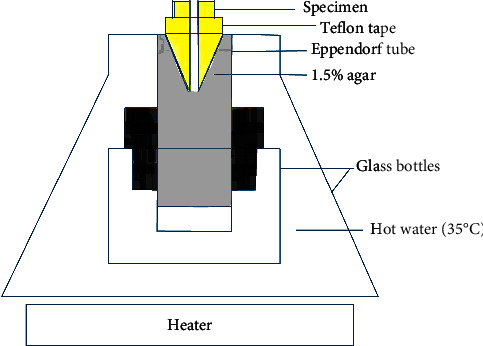
An illustration of the agar gel model in schematic form. An Eppendorf tube contains 1.5% agar gel and a tooth submerged into the gel. The equipment was mounted and secured within an opaque glass bottle. A glass container was used to simulate intracanal temperature while submerging the entire apparatus into 35°C hot water.

A modified version of the Lu et al. [19] approach was used to assess the amount of debris extruded apically. The teeth were then numbered after being divided randomly into seven groups (*n* = 20). The root surfaces of the specimens were covered with Teflon tape, leaving the apical foramen and root face exposed. The mean weight of samples was calculated after three consecutive readings of the weight of the samples using an electronic microbalance of 10^–5^ g (AUW-220D; Shimadzu, Tokyo, Japan). The Eppendorf tubes were filled with 3 ml of 1.5% agar gel before the samples were placed inside the tubes and secured with cyanoacrylate adhesives at the level of cementoenamel junction. After that, the tubes were inverted for the samples to be embedded into the agar. After the agar was gelated, three measurements of the weight of the tubes were taken. These measurements included the weight of the agar solution. The total weight of each apparatus (without the tooth apparatus) was determined by taking the difference between the first and second weight readings [16, 19, 20]. Eppendorf tubes were positioned in a brown glass bottle filled with water. In a hot water bath, the apparatus was placed at 35°C. (Figure 1, a schematic presentation of the agar gel model).

### 2.3. Root Canal Preparation

In the mesial canals of the teeth, a glide path was established using 25 mm-long pathFile (Dentsply Maillefer) instrument 1 (size 13, 0.02 taper) and 2 (size 16, 0.02 taper). Each root canal was prepared with a new file. Following three pecking movements in all groups, the file was removed from the canal, and the flutes of the instrument were cleaned with sterile gauze impregnated with alcohol. A 2 mL of distilled water was used to irrigate the canals at the removal of files, and a #10K-file was used to the working length for recapitulation; after recapitulation, the canal was irrigated again with 2 mL of distilled water. To standardize the preparation of the root canal and the quantity of the irrigant, we repeated these steps five times until the desired working length was attained, and 20 mL of distilled water was utilized. Root canal irrigation was performed using a 30-G side-perforated closed-ended needle (NaviTip, Ultradent, South Jordan, UT) positioned into the canal 2 mm shorter than the working length [16, 19, 20]. Canal patency was checked using a size 10K-file to the working length. A single operator expert in the instruments and techniques used performed all the root canal preparation procedures. VDW silver endodontic motor (VDW, Munich, Germany) was used in all instrumentation groups with the reciprocating type of file motion except for the OR group because of the compatibility matters of the type of reciprocation movements required for this group (170°CCW, 60°CW).

1. The WOG group: the WOG primary file (size 25, 0.07 taper) was used with the “WaveOne All” mode of a VDW silver endodontic motor (VDW, Munich, Germany). The manufacturer's instructions were followed, and the file was used with a slow pecking motion until the working length was reached. After three pecks, the instrument's flutes were cleaned.
2. Rec Blue group: in this group, the R25 file was utilized with the “Reciproc ALL” mode of the VDW silver endodontic motor (VDW, Munich, Germany). The manufacturer's instructions were followed, and pecking movements were performed with gentle strokes.
3. OR group: in this group, OR file (size 25, 0.06 taper) was used at the working length in a reciprocating motion (170°CCW, 60°CW), following the manufacturer's instructions, at 4 Ncm torque and a rotational speed of 400 rpm of an endodontic motor (AI-motor; Woodpecker, Guilin, China).
4. OC group: in this group, the OC file (size 25, 0.06 taper) was used at the working length following the manufacturer's instructions, at 2.5 Ncm torque and a rotational speed of 300 rpm of an endodontic motor (AI-motor; Woodpecker, Guilin, China).
5. HEDM group: The HEDM One File (size 25, 0.08 taper) was used in this group. Pecking movements and gentle apical strokes were applied; following the manufacturer's instructions, the files were coupled with the VDW silver endodontic motor (VDW, Munich, Germany) at a continuous rotational speed of 500 rpm and 2.5 Ncm torque.
6. Neoniti group: in this group, the A1 file (size 25, 0.06 taper) was used at the working length following the manufacturer's instructions, at 300 rpm rotational speed and 1.5 Ncm torque in VDW silver endodontic motor (VDW, Munich, Germany).
7. XPS group: in this group, the XPS file was used following the manufacturer's instructions at 800 rpm rotational speed and 1 Ncm torque in the VDW silver endodontic motor (VDW, Munich, Germany). Gentle strokes were applied until the desired working length was achieved. Upon reaching the working length, we performed the irrigation. The canal was again instrumented with gentle in and out movements for another 10 strokes to the working length, following the instructions from the manufacturer.

### 2.4. Data Collection and Statistical Analysis

Once we prepared the root canals, we took the Eppendorf tubes out of the glass bottles and the Teflon bands and teeth out of the Eppendorf tubes. We took three consecutive measurements of the weight of each apparatus. To calculate the amount of extruded debris, we subtracted the tooth-free apparatus's weight value from the postoperative weight value. Each tube that contained debris and distilled water had its mean weight measured. A second operator performed all measurements independently.

The amounts of debris extruded were statistically examined using IBM SPSS 28.0 software (IBM Corp, Armonk, NY). The assumption of the data's normality was verified using the Shapiro–Wilk test. Analysis was done by one-way analysis of variance and *post hoc* Tukey's tests. The significance level adopted was 5%.

## 3. Results

Table 1 describes the mean values and standard deviation of the amount of debris extruded apically in each experimental group. All the instruments caused debris extrusion beyond the apical foramen. Compared with the REC Blue, WOG, and OR groups, the XPS, OC, and Neoniti groups produced significantly less apically extruded debris (*p*  < 0.05). No significant difference was observed among the HEDM, WOG, OR, and REC Blue groups (*p* > 0.05). No significant difference was observed among the XPS, OC, Neoniti, and HEDM groups (*p* > 0.05).

## 4. Discussion

The null hypothesis was rejected, considering significant differences between the instrumentation systems used regarding the amount of apically extruded debris. Mesial roots of mandibular molars with curvatures ranging from 20° to 40° [17] were chosen due to the likelihood of a higher volume of extruded material during the preparation of curved canals compared with the straight canals. This is primarily attributed to the increased technical complexity of these conditions [21, 22, 23].

The instrumentation of the root canal system is frequently associated with postoperative pain and swelling. Cleaning and shaping procedures of the root canal may push the microorganisms, necrotic pulp tissues, dentin particles, and irrigants into the periradicular region [24]. Apical extrusion of debris is responsible for the acute inflammatory reaction that results from localized trauma and disordered balance between host defense and root canal microbiota [25]. Debris extrusion beyond the apex may also exacerbate inflammatory reactions [26]. This was investigated *in vivo* in further detail by Caviedes-Bucheli et al. [27], who concluded that the expressions of neuropeptides such as substance P (SP) and calcitonin gene-related peptides (CGRP) released from the human inflamed periodontal ligaments when stimulated by the apically extruded debris because of the effects of two single-file reciprocating root canal instrumentation systems (Reciproc and WaveOne [Dentsply Sirona Endodontic, Baillagues, Switzerland]) and the hand instrumentation. According to their results, the lowest expressions of SP and CGRP were discoverable in the group of teeth instrumented with Reciproc. The authors concluded that this difference could be ascertained by the various types of movement kinematics and the different design characteristics of the instrumentation system. Because quantifying apically extruded debris in vivo is not viable, we investigated the amount of apically extruded debris under laboratory conditions at body temperature using different single-file instrumentation systems.

Distilled water was chosen for irrigation to ensure that it would not impact the weight of the extruded debris [28, 29, 30, 31]. Using sodium hypochlorite could lead to errors in final weight readings because of the presence of salts [9].

In the present *in vitro* study, PathFiles were used to establish a glide path in all the groups. This facilitated the instrumentation in curved canals as per our inclusion criteria. In their clinical and experimental study, Kim et al. [32] concluded that the PathFile system is considerably safe and beneficial when establishing a glide path in extremely narrow canals. The amount of debris extruded apically during root canal preparation with seven new NiTi files (REC Blue, WOG, OR, HEDM, Neoniti, OC, and XPS) was investigated and compared for the first time. In our study, we used the modified model of the method described by Lu et al. [19]. We placed the apparatus used in this study in a 35°C hot water bath to mimic the clinical conditions of the same mean temperature of the root canal system, as reported by de Hemptinne et al. [33] and demonstrated by Uslu et al. [16].

The improved metallurgy, thermomechanical characteristics, and distinctive surface treatments of contemporary endodontic single-file instruments contribute to their augmented performance in endodontics [34]. REC Blue has two cutting edges and an S-shaped horizontal cross section, similar design features to the Reciproc. However, the new heat treatment of REC Blue has made the file more flexible and improved its structure [35, 36]. The file presents with a blue color as a result of the process of heat treatment [35]. WOG file has two cutting edges, improving fracture resistance and shaping efficiency [37, 38]. As indicated by the manufacturer, the cross section (parallelogram and two cutting edges), taper, and tip diameter were modified; hence, the instrument provides more flexible files as compared with earlier versions of WaveOne (Dentsply Tulsa Dental Specialties, Tulsa, OK, USA) [39]. Compared to the WaveOne primary file, the WOG primary file was found to have up to 50% higher resistance to the cyclic fatigue [13]. Compared to WaveOne and other instruments, Adiguzel and Caper discovered that WOG had greater resistance to torsional stress and cyclic fatigue [37, 40].

XPS has a fixed taper of 1% and an apical diameter of 0.30 mm. Because of the MaxWire alloy (FKG, Dentaire SA, La Chaux-de-Fonds, Switzerland) technology of XPS, it presents with the martensite phase at room temperature, which changes to the austenite phase at body temperature because of its subsequent “snake shape” taper of file increases from 1% to 4% [41]. HEDM and Neoniti files are manufactured using an electrical discharge mechanism with the controlled memory alloy [35, 42, 43, 44]. HEDM presents three types of horizontal cross sections associated with the working part of HEDM: the apical part has quadratic, and the middle and coronal parts have trapezoidal and triangular cross sections [42]. Because of the alloy's heating and controlled memory properties, it is shapeable. Thus, using them effectively and safely inside the root canal system is possible. The electric discharge machining method involved in the manufacturing of HEDM and Neoniti not only enhances the cutting abilities of the files because of the rough surface of the instruments, but it also has certain advantages such as limited manufacturing stresses to the file surface, creation of various designs without tool constraints, and high precision [43].

A heat treatment (C-wire) of OR files increases their centering ability and makes them more flexible. OR files should have considerable cutting efficiency because of their deep flutes and a cross section that is off-centered and variable that starts as a triple helix and changes progressively toward the shank as an S-shape. This design feature allows more amount of debris to evacuate coronally. OR files exhibit the reciprocating type of motion with specific angles as follows: 170° counterclockwise [CCW] and 60° clockwise [CW]. The later movement (CW) produces a cutting action. OC single-file system, though, works in a continuous rotation, but it has the same design characteristics as OR.

The instrument cross section is one of the key considerations in determining the amount of apically extruded debris. Due to the different cross-sectional shapes, more space between the file and the dentinal walls may allow more coronal flow of the dentinal debris. The triple helix and S-shape off-centered cross section of OR and OC allow the removal of much more debris from the root canal than the REC Blue, which has a horizontal shape with two cutting edges, and the parallelogram-shaped (WOG) which has an off-centered cross section with two cutting edges [15]. This may explain why more debris extruded apically by REC Blue than by other file systems investigated in our study.

Our results indicate that all instruments utilized in the present study generate apical extrusion of debris, which is consistent with the literature that all file systems produce apically extruded debris [3, 29, 45, 46]. The study's null hypothesis was rejected because the XPS generated significantly less debris than REC Blue, WOG, and OR. Our results are consistent with the other studies, which reported that the instruments with a reciprocation type of motion might produce more apically extruded debris than those with a continuous rotation motion [3, 10, 47, 48, 49, 50].

It is essential to mention that some studies reported different results related to the debris extrusion associated with reciprocating files. Boijink et al. [13] reported less debris extrusion with reciprocating instruments than with other techniques. De-Deus et al. [21] found that reciprocating instrumentation systems extruded significantly less debris than systems with full-sequence rotary files. Kocak et al. [51] found that reciprocating files produce less debris apically, but the difference among various experimental groups was not statistically significant.

In the present study, during root canal preparation, debris extrusion was observed, and no significant difference was found among HEDM, OR, WOG, and REC Blue instrumentation systems. These results are consistent with Uslu et al. [16] and Elashiry et al. [52], who studied debris extrusion and observed no significant difference between REC Blue and HEDM groups.

Different studies obtained different results on apically extruded debris because of differences in methodologies and the types of instruments used. A direct comparison among the instruments studied in the present study has not been made previously; hence, we cannot directly compare our results with other studies. Because of limitations related to studies done on extracted teeth, their results should be applied carefully in clinical situations. Periapical tissues around the apical foramen in clinical conditions may act as a barrier against the extrusion of debris and irrigants [53, 54]. Although floral foam can act as an artificial barrier [53], this method of simulation of periapical tissues is not without its limitations [55]. 1.5% agar gel is reported to have a density similar to the periapical tissues, as reported by Lu et al. [19] and utilized elsewhere in the literature [16, 20]. Therefore, we used agar gel to simulate the natural apical barrier. However, the agar gel model has some limitations, primarily because of the standardized thickness of the agar gel around the apex. Clinically, the extent of periapical disease may have caused apical bone loss or changes in the periodontal ligament [56]. Furthermore, differences in the dentin microhardness might affect the results when natural teeth are used, as reported by Tanalp and Güngör [57].

The apical extrusion of debris may result in postoperative pain and complications, and such complications are reported up to 16% in the literature [4]. Since the canal curvature and its anatomical variations in molar teeth increase the difficulty level of the instrumentation and influence the apical extrusion of debris [11, 12], this study holds significant clinical relevance as it will guide the operators in the chairside decision-making process based on the cases they have.

## 5. Conclusion

Within the limitations of the present study, all the instruments generated some extrusion of debris beyond the apex. The recorded amounts of debris that were generated apically for various files studied were REC Blue > OR > WOG > HEDM > OC > Neoniti > XPS. Compared with REC Blue, WOG, and OR groups, the generation of apically extruded debris was significantly lower in the XPS, Neoniti, and OC groups (*p*  < 0.05). No difference was found between the HEDM and any of the other NiTi files studied.

## Figures and Tables

**Table 1:** Weight of apically extruded debris in grams.

Group	Mean ± SD
Reciproc Blue^a^	0.0118 ± 0.0008
WaveOne Gold^ab^	0.0096 ± 0.0075
One Reci^ab^	0.0098 ± 0.0078
Hyflex EDM^abc^	0.0093 ± 0.0041
Neoniti^c^	0.0060 ± 0.0031
One Curve^c^	0.0061 ± 0.0028
XP-endo Shaper^c^	0.0058 ± 0.0039

Values with the same superscript letters were not statistically different (*p* > 0.05).

## Data Availability

The datasets analyzed during this study are not publicly available but are available from the corresponding author upon reasonable request.
